# Resilience in migraine brains: decrease of coherence after photic stimulation

**DOI:** 10.3389/fnhum.2012.00207

**Published:** 2012-07-24

**Authors:** Mayara Mendonça-de-Souza, Ubirakitan M. Monteiro, Amana S. Bezerra, Ana P. Silva-de-Oliveira, Belvânia R. Ventura-da-Silva, Marcelo S. Barbosa, Josiane A. de Souza, Elisângela C. Criado, Maria C. M. Ferrarezi, Giselly de A. Alencar, Otávio G. Lins, Maria das G. W. S. Coriolano, Belmira L. S. A. Costa, Marcelo C. A. Rodrigues

**Affiliations:** ^1^Grupo de Neurodinâmica, Departamento de Fisiologia e Farmacologia, Universidade Federal de PernambucoBrazil; ^2^Laboratório de Neurofisiologia Experimental, Fundação Municipal de Educação e Cultura de Santa Fé do SulBrazil; ^3^Neurofisiologia Clínica e Experimental, Departamento de Neuropsiquiatria, Universidade Federal de PernambucoBrazil

**Keywords:** migraine, EEG, partial directed coherence, human patients, resilience

## Abstract

**Background:** During migraine attacks, patients generally have photophobia and phonophobia and seek for environments with less sensorial stimulation. Present work aimed to quantify cortical partial directed coherence (PDC) of electroencephalographic (EEG) recordings from migraine patients and controls in occipital, parietal, and frontal areas with or without photic stimulation. Our hypothesis is that migraine patients with visual aura might have neuronal networks with higher coherence than controls even in interictal periods due to a predisposition in sensory cortical processing. **Methods:** Eleven adult women with migraine with visual aura (at least 48 h without previous attacks) and seven healthy adult woman were submitted to EEG recording in basal state and during photic stimulation. **Results:** When compared to healthy volunteers, migraine patients show different coherence profiles. Migraine patients had greater coherence than controls during the basal period (without photic stimulation), showing predisposition for sensory processing in many frequency ranges. After photic stimulation, patients showed a decrease in cortical coherence while controls had an increase. **Conclusions:** When compared to healty subjects, migraineurs show increased cortical coherence before photic stimulation, but a decrease when stimulation starts. This may be the expression of a resilience mechanism that allows migraineurs the interictal period. The PDC analysis permits to address a patient coherence profile, or “coherence map,” that can be utilized for management of the headache disorder or following up treatments.

## Introduction

Migraine is classified by the World Health Organization as the 19° most disabling disease. According to Stewart et al. (2008), four among 10 women and two among 10 men will have migraine in some moment in their lifes, most of them before 35 years. Despite its clinical importance, the pathophysiology of migraine has not been fully elucidated. Although being initially thought as only a vascular commitment (Camp and Wolff, 1961), migraine is nowadays considered as a neurovascular phenomenon characterized by frequent and recurrent attacks with a clear neurologic substrate even resembling, under certain circumstances, cortical spreading depression, and epilepsy (Sand, 1991; Rogawski, 2008). A typical attack is recognized by pain in a single half of the head, worsens with physical activity and is frequently associated with nausea, vomits, and distress to light and loud sound exposure, may taking up to 72 h (Bigal et al., 2009). Migraine attacks can be followed or preceded by an aura phenomenon, neurologic symptoms of cortical, or brainstem-cerebellar origin (Reinhard et al., 2012), that can be visual, sensory (e.g., facial or hand numbness), mental confusion, and disturbs of balance (Queiroz et al., 1997; Agarwal et al., 2012). Visual aura is the most common, and may present as bright scotomas, fotopsias, uni or bilateral hemianopsias, bright flashes, or colored lines (Queiroz et al., 1997).

Both migraine and some types of epilepsy can, under some circumstances, be induced by intermittent light stimulation (ILS) (Killam, 1979; Donnet and Bartolomei, 1997; Guerrini et al., 1997; de Tommaso et al., 1999; Goadsby, 2007). Therefore, ILS could be useful for unmasking neuronal networks possibly hyperactive and coherent in migraine or epileptic patients, compared to normal health volunteers; and for staging, following and predictive purposes. Migraine patients are hypersensitive to a constellation of stimuli, even when not within an attack (Bigal et al., 2009). A spontaneous migraine attack with simultaneous PET scanning showed a decrease in regional cerebral blood flow (rCBF) in the occipital region, and the headache was associated with bilateral hypoperfusion that started in the occipital lobes and spread anteriorly into the temporal and parietal lobes (Woods et al., 1994). Even in the interictal period, migraine patients have a visual cortex processing different from normal volunteers in deep brain and genetic scale (Vincent, 1998), and migraineurs showed hyperexcitability of the occipital cortex by functional magnetic resonance imaging-blood oxygenation level dependent (fMRI-BOLD) (Martín et al., 2011). There are evidences that brain has neuronal networks properties, hence resilience mechanisms, to avoid attacks. Migraine has gender selective incidence, with higher prevalence in female, and studies of brain networks showed. In female patients, brain functional networks showed worse resilience, more regions exhibited decreased nodal centrality, and more functional connections revealed abnormalities than in male patients (Liu et al., 2011).

The electroencephalography (EEG), a non-invasive and widely used methodology to record electrical activity from brain (Berger, 1933), is not generally used for clinical evaluation of migraine, as opposed to other disorders such as epilepsy, due to no identification of morphological landmarks by simple visual inspection and contradictory results (Sand, 1991). However, new mathematician and technical improvements have clearly shown that the so-called spectral analysis uncovered many interesting phenomena related to migraine. It was shown an increase of theta power in EEG recording of occipital regions in migraineours in the interictal period (Bjørk et al., 2009, 2011). But the EEG spectral parameter called partial directed coherence (PDC) could be interesting for the study of the neurophysiological basis of migraine since it describes if brain areas are influencing each other, if there is feedback and in what frequency oscillation it happens, based on the Granger theory of causality (Sameshima and Baccalá, 1999; Baccalá and Sameshima, 2001).

The aim of this study was to quantify the PDC between EEG recordings of occipital, parietal, and frontal cortical areas of migraineurs and health volunteers after photic stimulation, aiming differential connectivity among neuronal networks. Our hypothesis: after photic stimulation, migraineurs might show a stronger functional connectivity between occipital and adjacent regions due to predisposition and genetic background.

## Materials and methods

### Participants

Migraine with aura patients (female, *n* = 11, 19–45 years), and healthy volunteers (controls, females, *n* = 7, 19–45 years), underwent EEG recording. All patients were clinically diagnosed with migraine according to the International Headache Society Classification, and were having an average of three attacks weekly, varying from 1 to 3 h. Patients were free of attacks in the 48 h that preceded the recording day (interictal phase). The experiment was authorized by the Ethical Committee of Medicine School of São José do Rio Preto (FAMERP, process 140/2008) and by the Ethical Comitee from the Center of Health Sciences of Federal University of Pernambuco (UFPE, process 307/2011), and subjects gave their written permission for the use of their data for scientific and teaching purposes.

### Assessments

#### Electroencephalography

**Figure 1:**
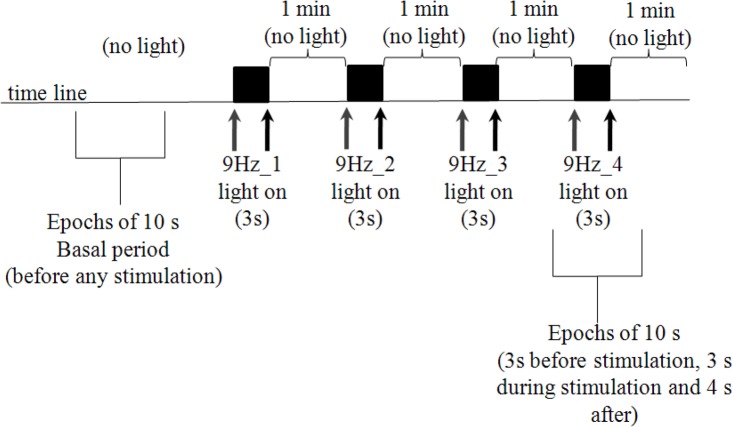
**Photic stimulation protocol.** Subjects remained with eyes closed during the whole experiment (that took approximately 11 min), to avoid eye blinking artifacts. Ten seconds epochs were taken before any stimulation occurs (basal period). Gray and black arrows indicate moments in which stimulation was turned on and off, respectively. Four trains of 9 Hz stimulation (3 s duration) were applied. In the present work, 10 s epochs including the basal and fourth stimulation period (9 Hz_4), were included in the analysis.

Subjects were submitted to EEG recording (Neurotec 40i or Neuro-Spectrum, international standard 10–20, low-pass 0.1 Hz and high-pass 200 Hz, sampling frequency 200 Hz) before (basal period) and during a 9 Hz photic stimulation. Photic stimulation was applied with the intensity of 0, 2 J, 20 cm from the subject's eyes, that remained closed during the experiment. The 9 Hz frequency was chosen because this frequency had a strong effect in synchronizing EEG recordings of occipital areas from migraine patients (Tommaso et al., 2007). The stimulator was programmed to deliver four trains (3 s each) of 9 Hz stimulation, that we called 9 Hz_1 to 9 Hz_4 (see Figure 1 for details). Artifact-free epochs of 10 s, including 3 s before stimulation, 3 s during 9 Hz stimulation and 4 s after light is off, were chosen for coherence analysis. In this work, it was analyzed only epochs within the 9 Hz_4 period. Also, 10 s of EEG recording were taken in the basal period (before any stimulation).

The PDC analysis was calculated as described by Sameshima and Baccalá (1999). In the present work it was analyzed the partial coherence between occipital (O1 or O2), parietal (P3 or P4), and frontal (F3 or F4) recordings for both hemispheres (odd numbers: left hemisphere). Partial coherence was computed within frequency bands: delta (1–4 Hz), theta (5–8 Hz), alpha (9 a 13 Hz), beta (14–29 Hz), and gamma (30–100 Hz) (Tatum, 2007). All neuronal networks were analyzed in diads, e.g., O1 to P3 (O1-P3), P3 to O1 (P3-O1) etc., and coherence values of basal and stimulation period were compared within the same hemisphere, using Mann–Whitney test, and the level of confidence was considered *p* < 0.05. Also, the evolution of coherence in time was averaged among controls and patients in the 9 Hz_4 epochs.

To test the influence of spurious and uncontrolled electric artifacts, two sets of patients were recorded in different places: five patients in a neurologic clinic near Santa Fé do Sul, São Paulo, Brazil and six patients in the Clinical Hospital of the Federal University of Pernambuco (HC-UFPE) in Recife, Pernambuco, Brazil. But for statistical purposes, all results were compared only in subjects recorded in the same place.

## Results

**Figure 2:**
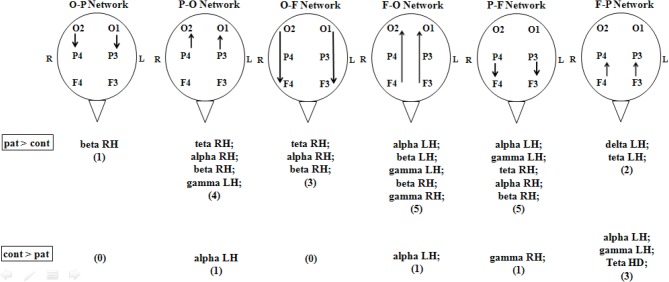
**Partial directed coherence of controls and migrainous patients in basal condition (no stimulation).** Patients had a general higher number of statistical significant coherence events when compared to controls, especially in parietal to frontal and frontal to occipital networks. Arrows indicate the direction of the network. O, occipital; P, parietal; F, frontal; cont, control health volunteers; pat, migraineurs patients. All events were considered different if *p* < 0.05, Mann–Whitney, between patients and controls.

In the basal period, that is before any photic stimulation occurs, it was clearly seen that migraine patients have increased coherence in many neuronal networks when compared to the control group, especially in parietal to frontal and frontal to occipital networks (Figure 2).

**Figure 3:**
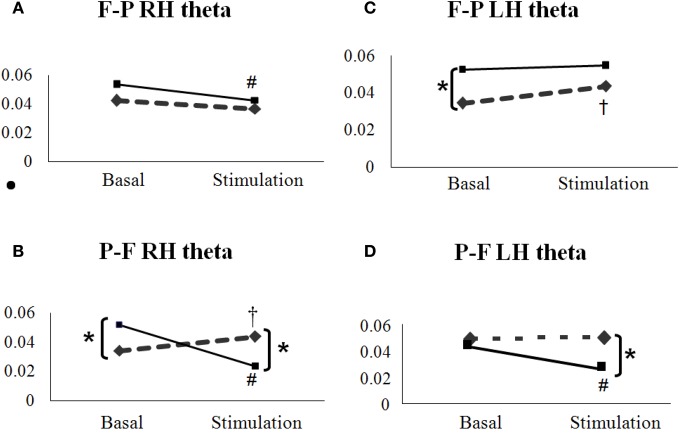
**Comparison between coherence values in healthy volunteers (diamonds, interrupted lines) and migraine patients (squares, solid lines) in EEG recordings from basal and photic stimulation epochs.** It is shown data from frontal and parietal networks, theta range. Controls generally present an increase of coherence after photic stimulation, as can be seen in P-F network **(B** and **C)**. Patients, however, have greater coherence values in basal period but with a strong tendency of decreasing with photic stimulation (**A,B**, and **D**). ^*^p < 0.05 between patients and controls; ^†^*p* < 0.05 between basal and stimulation in controls; ^#^*p* < 0.05 between basal and stimulation in patients; Mann–Whitney test was used for each comparison.

When photic stimulation starts, controls present an increase in coherence, as it could be expected since light is activating brain perceptual and sensory networks, especially those involving the occipital areas. But patients showed a decrease in coherence, especially right frontal to parietal (F-P) and parietal to frontal (P-F) in both hemispheres (Figure 3).

**Figure 4:**
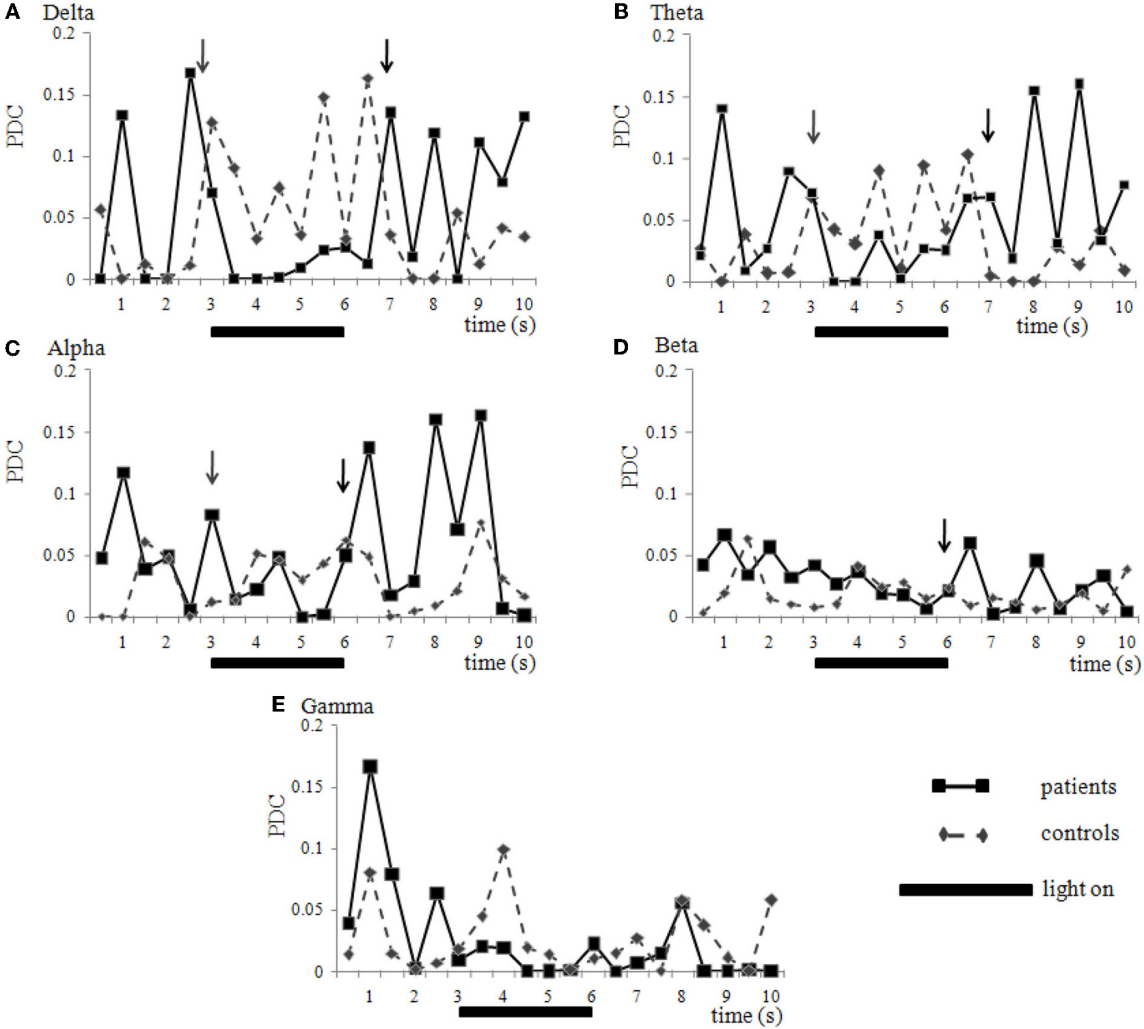
**Coherence variation in time.** Network O2-P4 (right hemisphere) in healthy volunteers (diamonds, interrupted lines) and migraine patients (squares, solid lines) during the 10 s epoch of EEG recording called 9 Hz_4. Photic stimulation was turned on during the 3rd and 6th second (solid bar). Data is presented as mean of partial directed coherence calculated from seven controls and five patients that were all submitted to the same stimulation and recording apparatus. Generally, controls present lower coherence than patients before stimulation and show an increase while light is turned on. However, patients present higher coherence before stimulation, with a decrease after lights are turned on (gray arrows), and increasing again, in peaks, after the stimulator is turned off (black arrows). This phenomenon may be seen in many frequency ranges like delta **(A)**, theta **(B)**, alpha **(C)** and beta **(D)**, but not necessary in all (e.g., gamma **(E)**, in this case) and neuronal networks analyzed. Statistical comparison between control and patient group is shown on Figure 3. Standard variations were suppressed for clarity.

When plotting the average of the PDC obtained from the EEG recordings of 7 controls and 5 patients during the 9 Hz_4 epochs in time, the effect of photic stimulation is clearly seen (Figure 4). Migraine patients generally have greater coherence than controls when the stimulator is off. When photic stimulation starts, there is a decrease in coherence in migraineurs and an increase in controls. After turning off the stimulator, patients tend to return to an increased coherence status while controls show a decrease (Figure 4).

This decrease of coherence in the 9 Hz_4 epoch compared to basal period was also seen in a supplementary group of six patients recorded in another equipment and different hospital (HC-UFPE, Recife). This decrease was seen in the networks P3-O1 (gamma range), P4-O2 (beta range), O2-P4 (alpha range), O2-F4 (delta range), and F4-O2 (beta) (data not shown), confirming the observed phenomenon.

## Discussion

Corroborant to our initial hypothesis, when compared to controls, patients show increased coherence before any stimulation (basal period). But when photic stimulation starts, there is a sustained decrease in coherence, especially in those networks involved in visual sensory processing occipital and parietal areas. This was interpreted as a resilience mechanism, that in spite of having a genetic and developed predisposition to migraine attacks, and these attacks are notable worsened by light, in the interictal period migraineurs can actually be exposed to some light up to a limit (that varies from one to one) without having an attack. It must be noted that the photic stimulation used in our protocol did not induce migraine attack in our patients (and controls). Our results corroborate at some extension what was found by Liu et al. (2011), where neuronal network properties in female predispose this gender to migraine attacks. To check if our results could be simply mathematical or EEGraphic artifacts, we performed the analysis twice, in two different clusters of patients, in different cities, operator, EEG setup equipment, and acquisition software. Results show that the specific neuronal network in which this decrease in coherence after photic stimulation occurs may vary from patient to patient, but the phenomenon is robust and was clearly seen in our data.

In a study with quantitative EEG analysis (QEEG), it was described a general increase in theta power in all cortical regions of migraineurs compared to non-migraine controls in a time free from pre-ictal symptoms (Bjørk et al., 2009), resembling what was found here. Despite a different approach, we could identify an increase of coherence too, but not exclusively, in theta frequency range of migraineurs compared to controls. It is possible that the increase in theta power described in that study could reflect a greater connectivity in this frequency range, but this can only be detected by PDC analysis.

We believe that the EEGraphic PDC measured reflects cortical interactions through direct neuronal cortical-cortical circuits or indirect (subcortical) loops (McHaffie et al., 2005). It is possible that the change in coherence observed here could be related to the neurophysiologic substrates of optical nerve stimulation, an effective procedure for medically intractable migraine (Weiner and Reed, 1999; Lambru and Matharu, 2012). Nerve stimulation has also being used in other diseases with altered brain sincronization, like vagal stimulation to treat epilepsy (Penry and Dean, 1990), and it is also described a change in coherence associated with seizure control (Warren et al., 2010).

A future approach of our work is to repeat the experiment identifying what are the most responsive neuronal networks in each patient, and following the evolution in coherence during a treatment to compare if clinical signals can be correlated to a change in brain coherence. If proved so, we imagine that this EEG analysis could be automated and be another tool for the clinician to readily categorize migraine patients within “EEG coherence maps” or profiles, establish the best approach and monitor treatment evolution. It is well-known that even those patients with similar clinical signals do not respond equally to treatments, so tools capable of identifying and categorizing patients (in this case, partial coherence) are desired.

### Conflict of interest statement

The authors declare that the research was conducted in the absence of any commercial or financial relationships that could be construed as a potential conflict of interest.
